# Neuromedin U mediates left atrial pressure–induced diuresis in the anaesthetised pig

**DOI:** 10.1371/journal.pone.0348773

**Published:** 2026-05-08

**Authors:** Vincent Healy, Therese Ruane O’Hora, Eric Lucking, Gerard O’Donoghue, Farouk Markos

**Affiliations:** 1 Department of Physiology, University College Cork, Cork, Ireland; 2 Biological Services Unit, University College Cork, Cork, Ireland; Ataturk University Faculty of Medicine, TÜRKIYE

## Abstract

Finding a potential biomarker for elevated left atrial pressure would be clinically useful as a diagnostic for pre-heart failure. Therefore, an investigation into whether the protein neuromedin U (NMU), which is significantly elevated in heart failure patients, mediates a classical diuresis that results from a sustained increase in left atrial pressure was conducted in the anaesthetized pig. Left atrial pressure was increased a little above 15 mmHg for 30 minutes in 8 chloralose anaesthetised female pigs. There was a significant increase in circulating NMU and urine flow rate, which surprisingly occurred without an accompanying natriuresis; both the serum NMU increase and the diuresis did not occur when the procedure was repeated post-vagal section. There was also a significant increase in glomerular filtration rate during the diuresis, which indicates a likely direct renal effect. The results show that NMU could be the mediator of the historic diuresis induced by an increased left atrial pressure. Future work to assess NMU levels in humans in pre-heart failure would be required to confirm NMU’s potential usefulness as a diagnostic.

## Introduction

An increase in left atrial pressure in experimental dogs was first shown to induce a large vagally dependent diuresis by Henry et al. [1,2]. Later studies aimed at detailing the firring patterns and location of the various atrial stretch receptors confirmed this diuresis and concluded that it was likely mediated by a centrally released blood borne agent, and was not ADH dependent [3,4]. The mediating agent was not identified, but a partial characterisation using plasma from this group indicated that the proposed compound had a molecular weight between 500–700 Daltons and lacked a disulphide bridge, both results rule out ANP as the cause [5].

A bioinformatics search using this and other information available indicated that only the protein neuromedin U (NMU) was close to meeting the known criteria for this novel purported diuretic. Subsequently pilot studies in our department showed that NMU injected into anaesthetized rats and pigs resulted in a rapid diuresis (unpublished observations, ongoing work). Therefore, the aim of the present study was to assess whether NMU levels in the circulation are increased in response to a sustained rise in left atrial pressure in vivo. This is potentially relevant clinically, since heart failure in humans is commonly preceded by an increased left atrial pressure [6,7] and so NMU could be a biomarker for this rise in advance of ventricular failure.

## Methods

### Ethical statement

All experiments were performed under licence approved by local and national ethical committees (HPRA, Dublin, Ireland). Project Number:AE19130/P124, Individual Authorisation number: AE19130/I167. All experiments were carried out under full general anaesthetic with a analgesic agent dose lasting longer than the surgical procedure and experimental protocol (doses are detailed in the general methods section). Humane endpoint was determined to be at the end of the experiment, 6–7 hours after initial sedation, where an overdose of KCL was given i.v. while under general anaesthesia and the heart and ecg were monitored to confirm death.

### General methods

Experiments were carried out on 8 female Landrace pigs weighing 24.25 kg (mean, range 22.5–27.07 kg). Pigs were sedated with ketamine (14 mg/kg i.m.) and xylazine (2.7 mg/kg i.m.) and then anaesthetized with *α*-chloralose via an ear cannula (induction 100 mg/kg), followed by the analgesic buprenorphine (0.02 mg/kg); maintenance chloralose (40 mg/kg/h i.v.) was infused via a central venous cannula inserted post induction and analgesia using an alarmed pump (B. Braun Dublin, Ireland). A continuous saline infusion (1 ml/min) was maintained during the entire experiment through a 3-way tap attached to the central line using a Harvard infusion pump (Holliston, MA, USA). A tracheotomy was performed and the pigs were ventilated with a mixture of 40% oxygen in room air using a ventilator (Harvard Apparatus). Expired carbon dioxide (PCO_2_), oxygen saturation (probe placed on the ear) and temperature were monitored using a Surgivet vital signs monitor (Surgivet, Smiths Medical, Dublin, USA). Core temperature, measured using a rectal or vaginal probe, was maintained at 37.5 ± 0.5°C by the heated surgical table (VSSI, Missouri USA). Arterial pH, PCO_2_ and PO_2_ were assessed using an i-STAT blood gas analyser (Abbot Point of Care Inc, Princeton, USA). A lead II ECG was attached, and a femoral artery cannulated to sample blood and to record systemic pressure using a short cannula connected to a pressure manometer (Grass, Quincy, U.S.A.). The accompanying femoral vein was also cannulated to sample blood.

### Surgery and instrumentation

**Fig 1:**
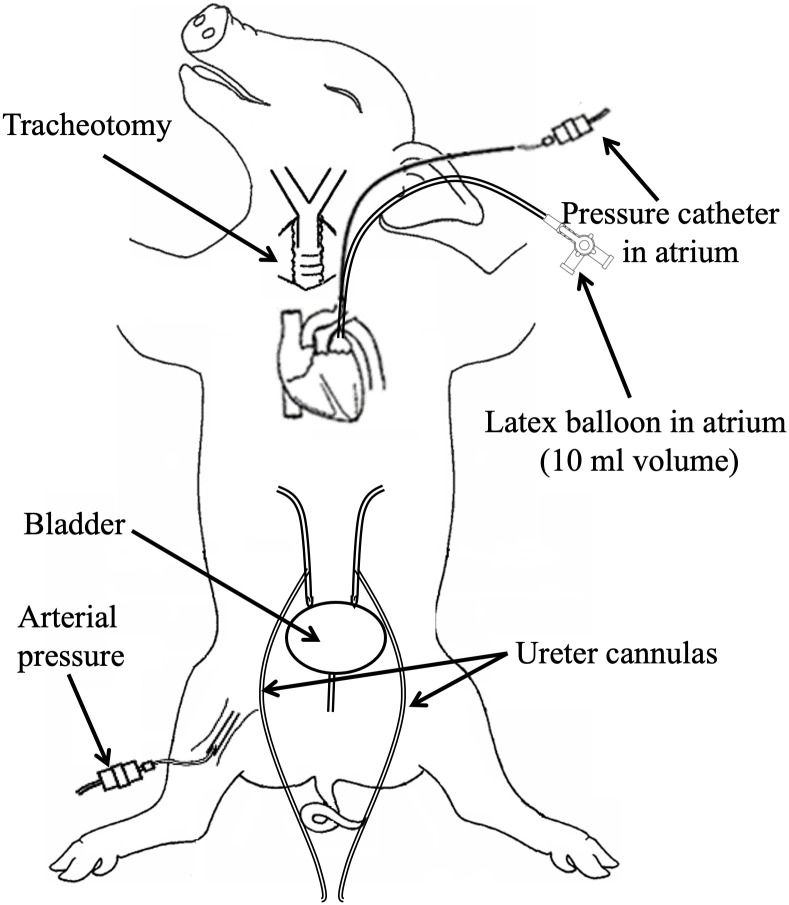
Schematic of the experimental preparation.

A schematic diagram of the preparation is shown in Fig 1. Briefly, a midline incision was made in the lower abdomen to expose the bladder, both ureters were dissected free and cannulated with 8F nylon catheters for urine collection. Then a sternal split was conducted and the chest was held open using rib spreaders, an incision (2–3 cm) in the pericardium was made and the left atrial appendage extracted. The appendage was stabilised and a small 1 cm cut was made into which a deflated 10 ml latex balloon (Harvard apparatus) connected to a cannula was inserted and advanced into the left atrium. A catheter tipped manometer (Millar) was also inserted into the left atrium using the same access for continuous direct measurement of left atrial pressure, both were tightly secured by ligature. All parameters were recorded using Power lab amplifiers, software (AD Instruments Ltd, Oxford, UK) and displayed on a monitor. Following experimental procedures, animals were killed using a lethal intravenous injection of anaesthetic and KCl.

### Experimental protocol

Once the balloon was in situ, it was inflated using saline until left atrial pressure reached a little over 15 mmHg where it was maintained for 30 minutes. The balloon was then deflated for 30 minutes, the cervical vagi sectioned and a second 30 minute inflation was carried out followed by a 10 min deflation before the pigs were euthanised. During this protocol, urine and blood samples (3 ml) were taken, measured and prepared for analysis at 10 min intervals. Parameters assessed immediately in the laboratory were: urine flow rate; blood sodium, potassium and creatinine. Urine was assessed for sodium and creatinine levels independently in the laboratory of VPG (Unit 1 Brushville House, Dosco Business Park, Cork) using a Beckman Au-480 analyser.

Serum was prepared in the lab by using BD Vacutainer Rapid Serum tubes (orange caped, Northwest Business Park, Dublin). As per manufacturer instructions, samples were added to these tubes and left to stand for 5 minutes prior to centrifugation using a Medifuge centrifuge (Fisher Scientific, Dublin) at 5000 rpm (approximately 3500–4000 g) for 5 minutes. 1–1.5 ml of serum was then collected after centrifugation using Pasteur pipette and transferred to a labelled Eppendorf tube, samples were frozen for later NMU analysis.

### NMU assessment

A pig Neuromedin U-25 ELISA kit was obtained from LifeSpan BioSciences Inc., Newark, California, United States. All serum samples, diluted 1:10 with ELISA buffer, were assayed in triplicate according to manufacturer’s instructions. Standard curves, generated from NMU-25 standards provided with the kit, were used to calculated serum NMU concentrations. The assay had a lower limit of detection of 0.03 ng/mL and a recovery rate of 92.8%. The intra-assay and interassay coefficients of variation were 5.3% and 7.1%, respectively.

### Statistical analysis

Repeated measures ANOVA (SPSS) was used and P < 0.05 was taken to be significant. Data is presented as mean ± sem unless otherwise stated.

## Results and discussion

Baseline parameters with the chest open, before balloon inflation were: heart rate 107 ± 10 beats/min (range: 76–150), mean arterial pressure 72 ± 4 mmHg (range: 58–90), mean left atrial pressure 6 ± 1 mmHg (range: 2–9), urine flow rate 1.14 ± 0.11 ml/min (range: 1–2).

**Fig 2:**
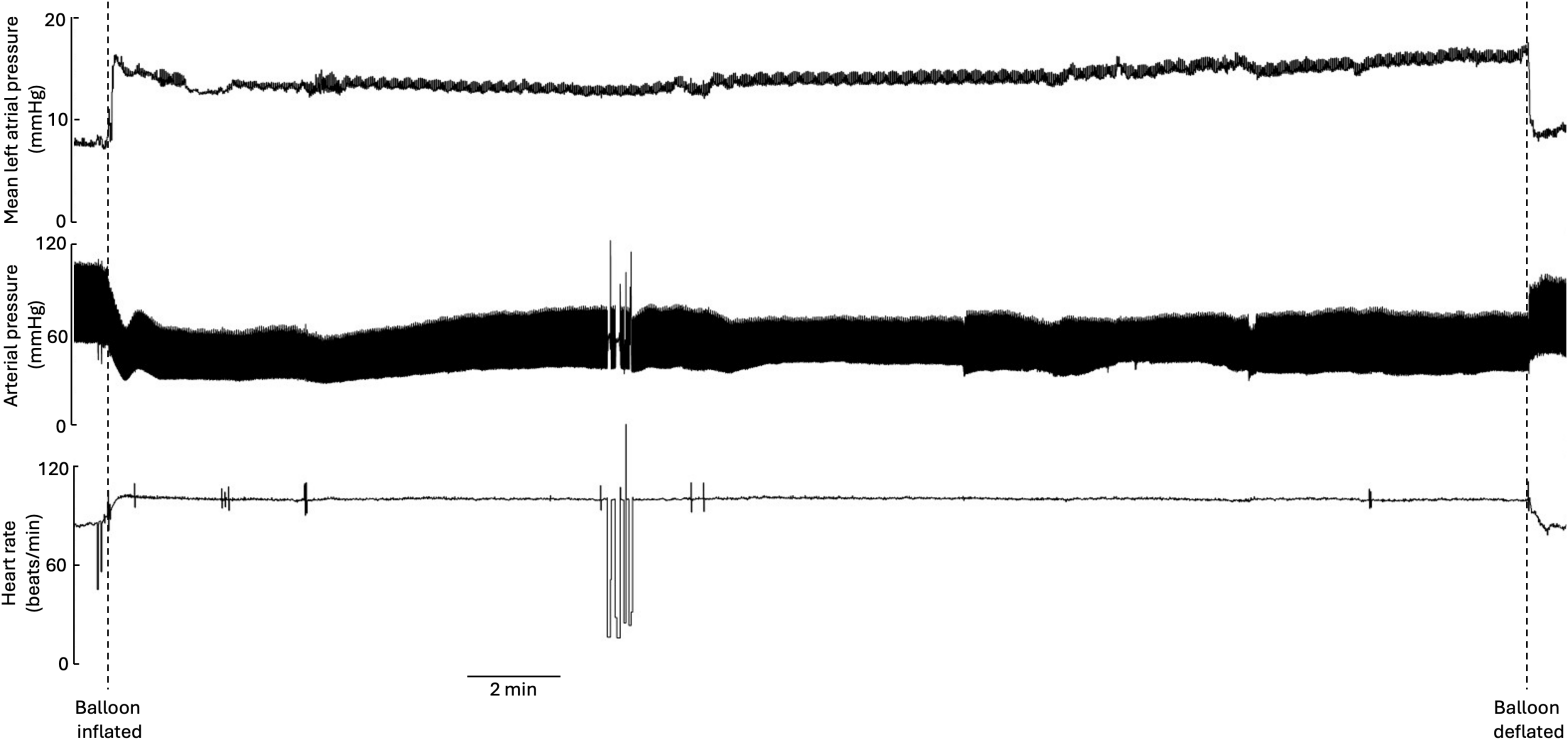
An example of records obtained from pig3/22 showing the increase in left atrial pressure with the vagi intact induces a fall in arterial pressure and a subsequent increase in heart rate which are both reversed after the atrial balloon is deflated. Blood and urine samples were collected at 10 minute intervals during this procedure and throughout the remainder of the experiments.

Fig 2 shows an example of records obtained from one balloon inflation-increased left atrial pressure experiment.

**Fig 3:**
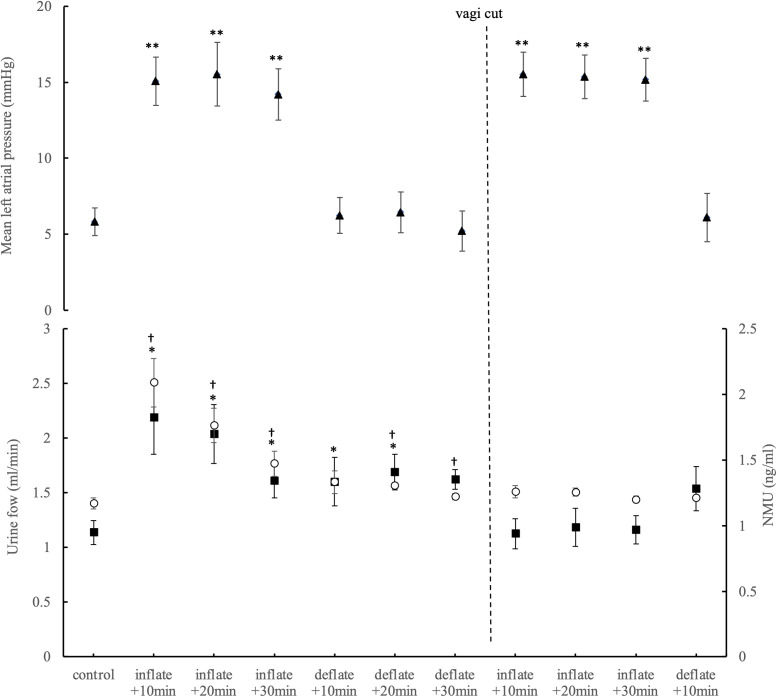
Increasing left atrial pressure causes a significant increase in both urine flow rate and circulating NMU which is absent when atrial pressure is increased following vagal section. Second graph, open circles show the NMU data with the scale on the right y-axis, closed boxes show the urine flow data with the scale on the left y-axis. Numbers on x-axis denote time in minutes following each inflation or deflation of the balloon in the left atrium. *P < 0.05 for NMU data compared to its control, †P < 0.05 for urine flow rate compared to its control, **P < 0.01; repeated measures ANOVA, n = 8 for all.

Data from all 8 pigs is summarised in Fig 3. There is a rapid increase for both urine flow and circulating NMU 10 minutes after the increase in left atrial pressure, with a slight fall from the peak that remains significantly higher for the full 30 minute inflation period. Urine flow is still elevated after left atrial pressure returns to normal, the rebound increase in arterial pressure after balloon deflation likely contributes to this (see the end of the arterial blood pressure record in Fig 2). It should be noted that urine output is increased despite the fall in mean arterial pressure during the balloon inflation period.

Further, this increase in both urine flow and circulating NMU does not occur when left atrial pressure is increased a second time post vagal section. In fact, urine flow for each 10 minute time points is significantly lower than the urine flow rate immediately prior to balloon inflation, in contrast to the first inflation protocol. This shows that this diuretic response is a reflex that requires intact vagi, and it confirms that the mediator is not released from the heart or periphery.

Table 1 summarises the data from all 8 pigs showing that there was a significant rise in eGFR in response to the increase in left atrial pressure due to balloon inflation without a concomitant increase in urinary sodium.

**Table 1:** Increased left atrial pressure causes a significant increase in eGFR without an accompanying natriuresis.

	Urine sodium (mmol/L)			eGFR (ml/min)		
	Control	Inflate	Deflate	Control	Inflate	Deflate
Pig 5/21	85	45	67	40	63	59
Pig 3/22	28	23	28	41	34	36
Pig 4/22	59	62	72	53	62	57
Pig 10/22	37	31	29	32	65	48
Pig 11/22	62	41	45	38	66	41
Pig 5/23	79	88	88	36	39	30
Pig 6/23	63	75	83	38	36	42
Pig 7/23	58	66	64	23	61	38
Mean	59	54	60	37	53	44
SD	19.30	22.54	22.99	8.37	14.21	10.11
SEM	6.82	7.97	8.13	2.96	5.02	3.57
n	8	8	8	8	8	8
P		0.360	0.981		0.035*	0.096

*P < 0.05 repeated measures ANOVA, data compared to respective control value.

The principle finding of the current study is that serum NMU is increased in response to a sustained rise in left atrial pressure which also results in a significant reflex diuretic response in the pig similar in character to the historic experiments in dogs. Circulating NMU was unchanged when left atrial pressure was elevated a second time after vagal section. It is important to highlight and emphasise that the diuresis occurs despite a fall in systemic blood pressure, since urinary output is dependent on mean arterial pressure. Also, there was a significant rise in glomerular filtration rate (eGFR) which suggests a direct effect on the kidneys and most interestingly this was not accompanied by a natriuresis. One of the foundations of this current study was the finding that exogenous NMU injected or infused into both rats and pigs in vivo results in a diuresis (unpublished observations). These results support the preliminary data and ongoing work using a PKA assay on the M1 collecting duct cell line indicate that NMU treatment blocks the activation of PKA induced by ADH, which would be predicted to result in increased water excretion by preventing aquaporin insertion in the collecting duct. Essentially the suggestion is that NMU induces water loss by blocking the effect of ADH in the latter nephron.

The lack of an increase in urinary sodium with the diuresis contradicts previous dog work where a natriuresis was only seen when left atrial pressure was increased, in contrast to the data presented here. In the classical dog experiments, stimulation of stretch receptors in the atrial appendage and pulmonary venous junction regions which did not raise atrial pressure gave a diuresis without a natriuresis [4]. Whether this contradiction is a technical measurement or species issue is not clear, what is known is that pig’s renal physiology does differ slightly from humans. For example, pigs have fewer nephrons and a lower glomerular filtration, in addition, pig ADH contains lysine instead of arginine which all have been highlighted as potential issues for kidney function post xenotransplantation [8]. The control eGFR values obtained are within the normal range for pigs [9]. The choice of raising atrial pressure to around or slightly above 15 mmHg is based on the observation that a pressure above the physiologically normal range of 6–12 mmHg is a reasonable index of heart failure [10]; if left atrial pressure rises above 25 mmHg, pulmonary oedema is known to occur [11].

NMU levels are significantly elevated in serum from patients in heart failure [12], as mentioned an increased left atrial pressure typically precedes heart failure and if detected could be diagnostic [13,14]. The control levels of NMU seen in the present study in pigs are at the lower end of the range of NMU in healthy humans [15,16]. However, the increased levels are far below those seen in heart failure [12]. Again, this could be due to a species difference, but another possible reason is that the pig model used employs imposing a rapid acute pathology or dysfunction on otherwise healthy animals. Which is different to the clinical aetiology of human disease.

## Conclusion

The data obtained supports the theory that NMU release is increased by a sustained rise in left atrial pressure and could in fact directly account for the associated diuresis. This latter assertion as well as the direct cardiovascular and renal effect of applied NMU is currently being investigated in our laboratory. Future work aimed at investigating whether NMU levels are higher in humans prior to the incidence of heart failure are planned.

## Supporting information

S1 Data Contains all data. (XLSX)
